# On Some New Hypnotics

**Published:** 1887-01-01

**Authors:** S. G. Webber


### ON SOME NEW HYPNOTICS.

By Dr. S. G. Webber.

Several drugs have been lately given to the profession with the claim that they are valuable hypnotics. I have had an opportunity of testing these in the cases of many patients in the Adams' Nervine Asylum.

*Paraldehyde* has been some years in use. It has advantages over chloral. The immediate subsequent effects are less unpleasant. It very rarely causes headache on the next day; sometimes patients have a sensation of fulness or pressure in the head for a few hours after waking. I gave it in one case where the patient had been taking chloral in large doses, not measuring the dose, and had been injuriously affected by the drug. The paraldehyde gave, in this instance, better and more prolonged rest; the patient partially recovered his mental powers and some measure of strength. The paraldehyde was continued several weeks in nightly doses of forty minims. In this patient there was probably disseminated sclerosis, and it did not seem possible to do without some agent to produce sleep, at least so long as he was at home.

The chief objection to paraldehyde is its disagreeable odor and taste, and also the odor it imparts to the breath. I do not remember to have met any unpleasant symptom arising from its use; but had never seen a case in which it has been used habitually for a long time. The dose is from forty-eight to eighty or ninety minims; sixty minims is the average dose. Occasionally a dose of eighty minims has failed to give the sleep which I have desired to obtain.

*Urethane* came into notice later than paraldehyde; it is much more pleasant to administer, having scarcely any taste or odor.

When I first gave it the dose was too small, but by increasing it to thirty grains good results were generally obtained. Sometimes much larger doses are necessary. It is quite soluble; fifteen grains will dissolve in a drachm of water.

The after effects are generally unimportant. It has seemed once or twice to give rise to nasuea the day after; but often the patient says the sleep has been very natural and refreshing. It is not so sure to produce sleep as paraldehyde.

*Hydrobromate of hyoscine* has the advantage of being almost tasteless, and the dose is small. I began with a dose of one hundred and twentieth of a grain, as recommended by the articles I had read, but soon found this too small, and increased to a sixtieth of a grain, using the following formula, giving twenty minims:

℞. Hydrobromate of hyoscine....................gr. j.

Alcohol.....................................ʒ iss.

Water sufficient for........................ʒ xx. M.

In a few instances I have given twenty-five minims of this mixture.

There is so little taste to this that it can be given in some simple drink, as gruel or beef tea, without the knowledge of the patient.

In one patient it produced discomfort in the head the next day, so that after two trials it was given up and urethane given instead. In other cases the sixtieth of a grain given for a week or more has produced no bad effects. In a few cases it has failed to produce sleep, and the sleep secured in other cases has not been of so long duration as that usually produced by paraldehyde, but subsequently in these same case the latter drug has not proved more efficacious. On the whole, my experience with this drug is that it will act favorably with a large number of patients, that it is acceptable on account of its lack of taste and odor, and because of its small dose; the sleep obtained is very refreshing and natural; it is, however, rather more likely to leave unpleasant effects, and seems to loose its power by repetition sooner than either urethane or paraldehyde.

*Hypnone* has a strong odor of bitter almonds. I have given it in capsules in doses of five to eight drops (.07 to .13) or even more, the former dose is rather small for good results. It will produce very natural sleep, and the patient awakes refreshed. It is of much less value than the drugs already considered, and fails more frequently than the others in producing sleep. It may be conveniently substituted for the others when they have been taken some time consecutively.—*Boston Med. and Surg Journal.*

